# Supplementary material for: Antibodies from the Sera of Multiple Sclerosis Patients Efficiently Hydrolyze Five Histones
Source: Biomolecules. 2019 Nov 15;9(11):741. doi: 10.3390/biom9110741 (PMC6920934; doi:10.3390/biom9110741)
Supplement: Supplementary file 1 [file biomolecules-09-00741-s001.pdf]

Supplementary Table 1. Several different characteristics of MS patients

№	MS subtypes	Sex	Age (years)	Disease duration (years)	EDSS*
Debut of multiple sclerosis (DMS)**					
1	DMS1	F	26	0.083	3.0
2	DMS2	F	24	0.17	4.5
3	DMS3	F	21	0	2.0
4	DMS4	M	30	0	3.5
5	DMS5	M	29	0	3.5
6	DMS6	F	20	0.083	1.5
7	DMS7	F	26	0.083	2.0
8	DMS8	F	43	0	4.0
Average values	8 patients	6 F and 2 M	27.4 ±7.2	0.05±0.06	3.0±1.1
Remitting multiple sclerosis (RMS)					
9	RMS1	F	21	1	1.5
10	RMS 2	M	46	16	4.0
11	RMS3	F	22	1	3.5
12	RMS4	F	53	23	3.5
13	RMS5	F	38	12	3.0
14	RMS6	F	58	20	3.0
15	RMS7	M	38	9	4.0
16	RMS8	F	45	11	4.0
17	RMS9	M	52	3	2.0
18	RMS10	F	34	1	3.5
19	RMS11	F	37	7	1.5
20	RMS12	F	30	14	2.0
21	RMS13	F	24	5	1.5
22	RMS14	M	31	5	1.0
23	RMS15	F	40	6	1.0
24	RMS16	F	55	10	3.5
25	RMS17	M	36	1	1.0
26	RMS18	F	36	4	3.0
27	RMS19	F	44	5	1.5
28	RMS20	M	35	3	1.0
29	RMS21	F	26	1	1.0
30	RMS22	M	23	4	1.5
31	RMS23	M	42	8	6.0
32	RMS24	F	40	17	6.0
33	RMS25	F	40	5	1.0
34	RMS26	M	51	12	3.5
35	RMS27	M	27	6	3.0
36	RMS28	F	30	8	1.5
37	RMS29	M	44	9	2.0
38	RMS30	F	40	1	2.5
39	RMS31	M	25	12	1.0
40	RMS32	F	46	7	3.5

41	RMS33	M	36	2	3.0
42	RMS34	M	43	1	6.0
43	RMS35	F	32	1	1.5
44	RMS36	M	30	1	1.5
45	RMS37	M	30	3	4
Average values	37 patients	21 F and 16 M	37.3 ±9.6	6.9 ±5.8	2.6 ±1.5
Secondary progressive multiple sclerosis (SPMS)					
46	SPMS1	F	45	2	7.0
47	SPMS2	F	43	1.5	4.0
48	SPMS3	F	23	3	2.0
49	SPMS4	F	37	6	3.0
50	SPMS5	M	33	10	3.5
51	SPMS6	F	48	17	4.5
52	SPMS7	F	33	7	1.5
53	SPMS8	F	55	12	6.5
54	SPMS9	F	24	2	1.5
55	SPMS10	M	41	7	1.0
56	SPMS11	F	32	1	1.0
Average values	11 patients	9 F and 2 M	37.6 ±9.9	6.2 ±5.1	3.2 ±2.1
Remittently progressive multiple sclerosis (RPMS)					
57	RPMS1	M	33	16	5.5
58	RPMS2	M	53	13	3.5
58	RPMS3	F	32	5	1.5
Average values		1 F and 2 M	39.3±11.8	11.3 ±5.7	3.5 ±2.0
Average Values	59 patients	38-F и 21 M	36.2±10.0	6.1 ±5.8	2.8 ±1.6

*Kurtzke's expanded disability status scale (EDSS)

**Debut of multiple sclerosis (DMS) corresponds to the first coming of patients in the clinic for research after the early manifestations of signs of this pathology
